# Supplementary material for: Prestige and dominance-based hierarchies exist in naturally occurring human groups, but are unrelated to task-specific knowledge
Source: R Soc Open Sci. 2019 May 1;6(5):181621. doi: 10.1098/rsos.181621 (PMC6549959; doi:10.1098/rsos.181621)
Supplement: Supplementary research materials [file rsos181621supp1.docx]

**SUPPLEMENTARY MATERIAL**

1. Wider-community questionnaires: pp.2-3
2. Quiz: pp.4-14
3. Within-group questionnaires: p.15
4. List of participating groups: p.16
5. List of named influential community members: p.17-20
6. List of named community learning models and learning topic p.21-24

**YOUR ID NUMBER:________ YOUR GENDER: ______ YOUR YEAR OF BIRTH: _______**

Please name someone who you think of as having **high status, or as being highly influential**, in either your **local community** or in **your country**. By “highly influential” we mean: is paid attention to, leads decisions. E.g. The Prime Minister, an M.P., Councilor, Mayor, or local celebrity:

Name: ___________________________ Their role/position/job: _________________________

Please rate this person according to the following scale:

**1------------2------------3------------4------------5------------6------------7**

**Very Much/ TRUE**

**Not At All/ NOT TRUE**

**Somewhat**

1. Members of your community respect and admire them _____
2. Members of your community do NOT want to be like them_____
3. They enjoy having control over other members of the community_____
4. Members of your community always expect them to be successful_____
5. They often try to get their own way regardless of what others in the community may want_____
6. Members of your community do NOT value their opinion_____
7. They are willing to use aggressive tactics to get their way_____
8. They are held in high esteem by members of the community_____
9. They try to control others rather than permit others to control them_____
10. They do NOT have a forceful or dominant personality_____
11. Members of the community know it is better to let them have their way_____
12. They do NOT enjoy having authority over other members of the community_____
13. Their unique talents and abilities are recognized by others in the community_____
14. They are considered an expert on some matters by members of the community_____
15. Members of your community seek their advice on a variety of matters_____
16. Members of your community are afraid of them_____
17. Others do NOT want to spend time with them_____

**YOUR ID NUMBER**: __________

Please name someone either from your country or your local community that you would like to **learn from** or **learn to be like**. E.g. learn a skill of theirs, or gain knowledge/advice from:

Name: ___________________________ What of theirs would you like to learn:_________________________

Please rate this person according to the following scale:

**1------------2------------3------------4------------5------------6------------7**

**Very Much/ TRUE**

**Not At All/ NOT TRUE**

**Somewhat**

1. Members of your community respect and admire them _____
2. Members of your community do NOT want to be like them_____
3. They enjoy having control over other members of the community_____
4. Members of your community always expect them to be successful_____
5. They often try to get their own way regardless of what others in the community may want_____
6. Members of your community do NOT value their opinion_____
7. They are willing to use aggressive tactics to get their way_____
8. They are held in high esteem by members of the community_____
9. They try to control others rather than permit others to control them_____
10. They do NOT have a forceful or dominant personality_____
11. Members of the community know it is better to let them have their way_____
12. They do NOT enjoy having authority over other members of the community_____
13. Their unique talents and abilities are recognized by others in the community_____
14. They are considered an expert on some matters by members of the community_____
15. Members of your community seek their advice on a variety of matters_____
16. Members of your community are afraid of them_____
17. Others do NOT want to spend time with them_____

**ART QUIZ**

1. **Henri Matisse was a French artist of the:**
   1. **15^th^ Century**
   2. **20^th^ Century**
2. **The singing butler is a famous modern painting by the Scottish painter:**
   1. **Jack Vettriano**
   2. **Andrew Geddes**
3. **The starry night is a famous painting by:**
   1. **Vincent van Gogh**
   2. **Jackson Pollock**
4. **The Birth of Venus**
   1. **Sandro Botticelli**
   2. **Leonardo da Vinci**
5. **Rembrandt was famous for which style of painting?**
   1. **Baroque**
   2. **Surrealism**


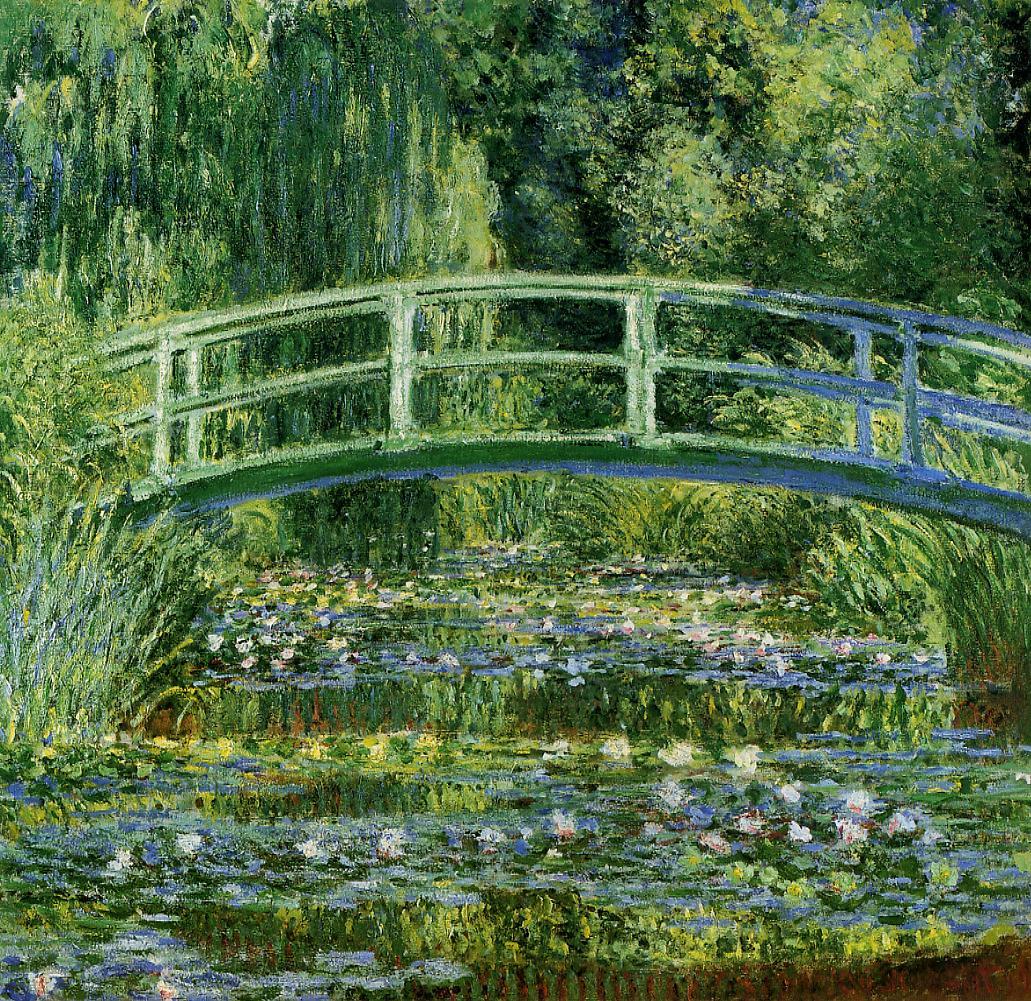


1. **The above is an image of a painting by which artist?**
   1. **Claude Monet**
   2. **Eduard Manet**


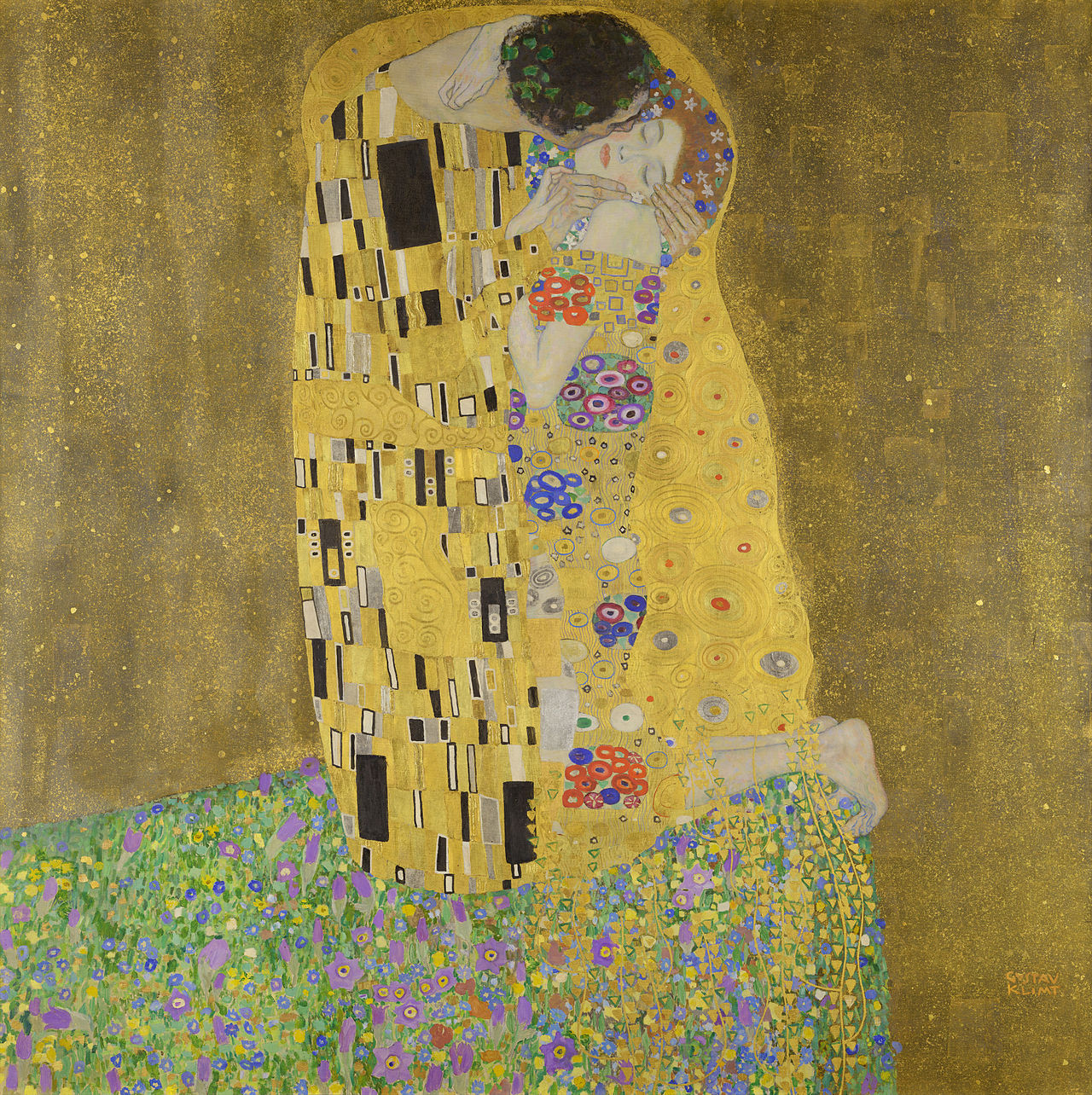


1. **The above is an image of a painting by which artist?**
   1. **Rudolf Hausner**
   2. **Gustav Klimt**

**
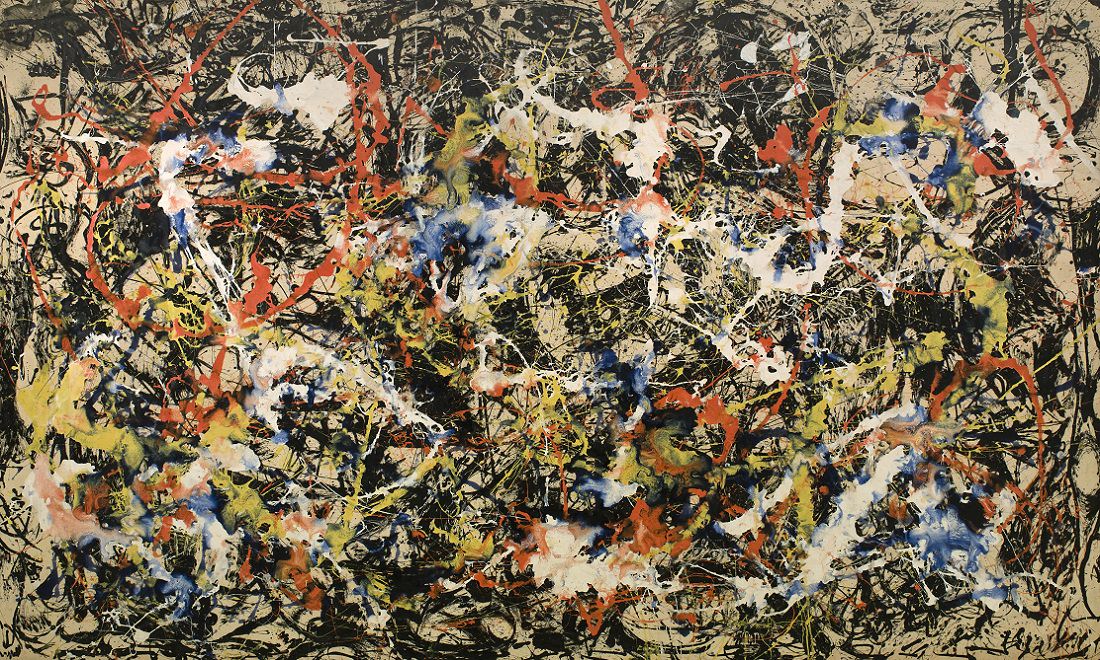
**

1. **The above is an image of a painting by which artist?**
   1. **Jackson Pollock**
   2. **Franz Kline**

**
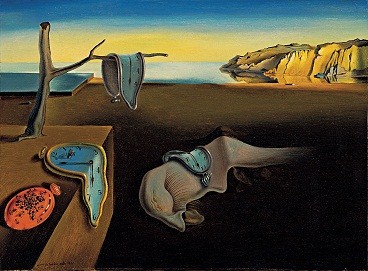
**

1. **The above is an image of a painting by which artist?**
   1. **Marcel Duchamp**
   2. **Salvador DalÌ**

**
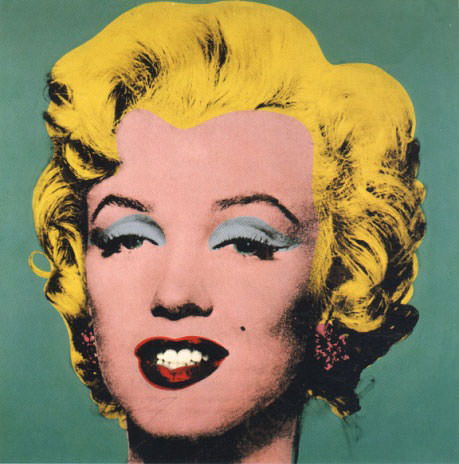
**

1. **The above image is associated with which artist?**
   1. **Roy Lichtenstein**
   2. **Andy Warhol**

**GEOGRAPHY QUIZ**

1. **Oklahoma state shares a border with:**
   1. **New Mexico**
   2. **Arizona**
2. **Tokyo is closer to:**
   1. **Hamamatsu**
   2. **Kyoto**
3. **The capital of the Philippines is:**
   1. **Davao**
   2. **Manila**
4. **Melbourne is in:**
   1. **Australia**
   2. **New Zealand**
5. **Which city is closer to Rome?**
   1. **Naples**
   2. **Florence**

**
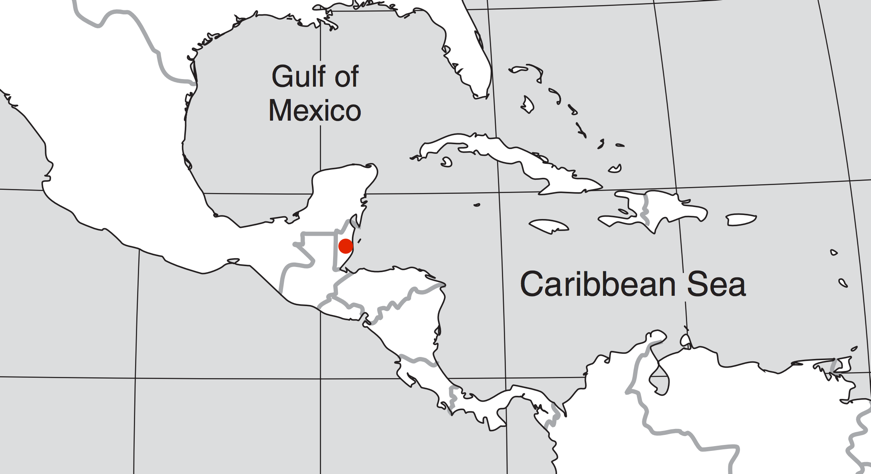
**

1. **In which country is the red dot located in? (Above)**
   1. **Belize**
   2. **Guatemala**

**
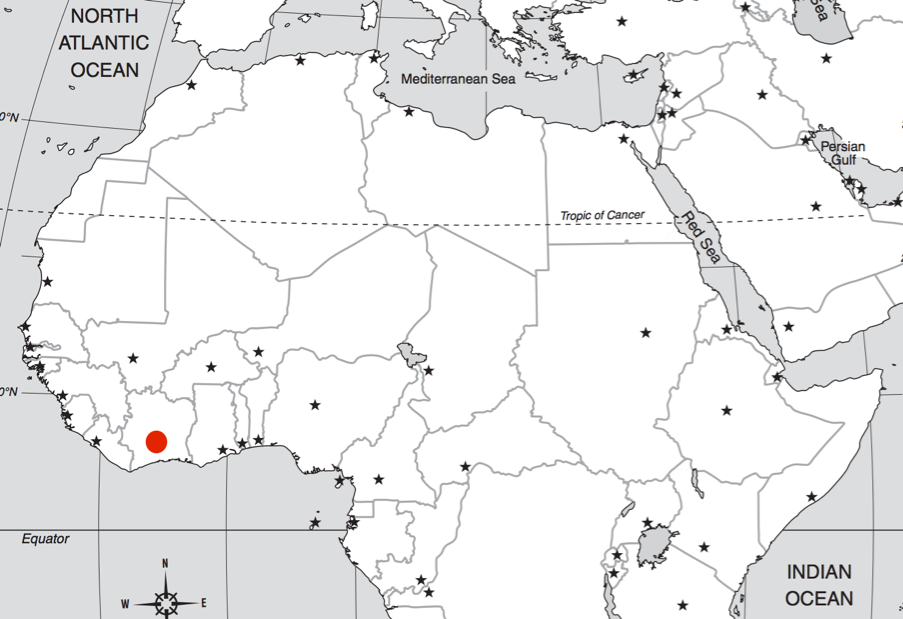
**

1. **In which country is the red dot located in? (Above)**
   1. **The Ivory Coast**
   2. **Nigeria**

**
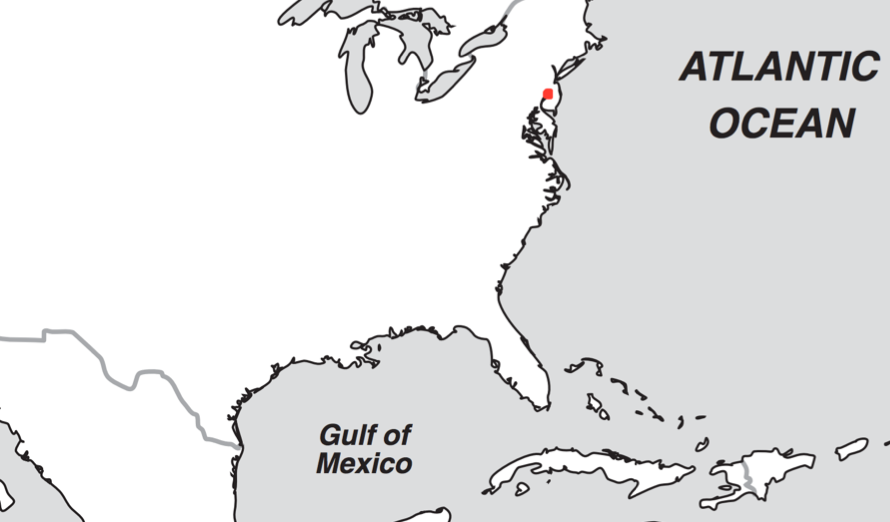
**

1. **In which city is the red dot located? (Above)**
   1. **Seattle**
   2. **Philadelphia**

**
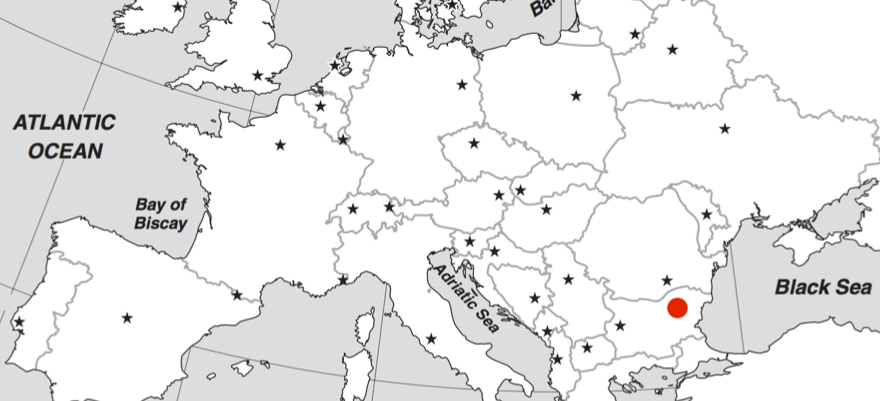
**

1. **In which country is the red dot located? (Above)**
   1. **Bulgaria**
   2. **Greece**

**
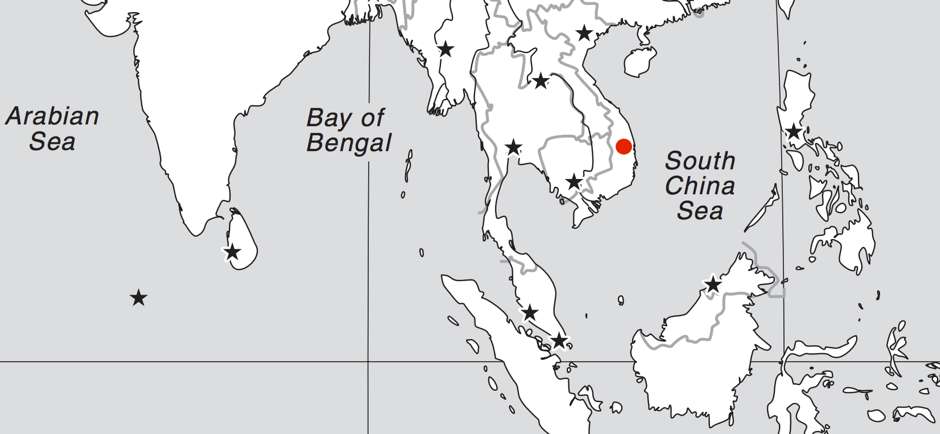
**

1. **In which country is the red dot located? (Above)**
   1. **Vietnam**
   2. **Laos**

**LANGUAGE IDENTIFICATION**

1. ***‘Gobierno’* means ‘government’ in which language?**
   1. **Spanish**
   2. **Portuguese**
2. ***‘Vinaka’* means ‘Thank you’ in:**
   1. **Fijian**
   2. **Swedish**
3. ***‘Umut’* is ‘hope’ in:**
   1. **German**
   2. **Turkish**
4. ***‘Pudel’* means ‘bottle’ in:**
   1. **Bulgarian**
   2. **Estonian**
5. ***‘Bloem’* means ‘flower’ in:**
   1. **Dutch**
   2. **Danish**

**
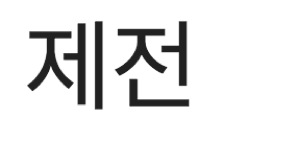
**

1. **The above means 'festival' written in which language?**
2. **Japanese**
3. **Korean**

**
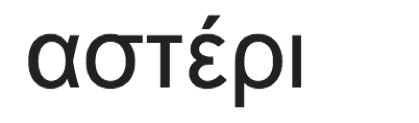
**

1. **The above means ‘star’ written in which language?**
   1. **Macedonian**
   2. **Greek**

**
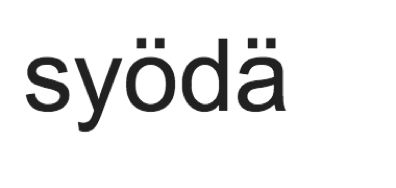
**

1. **The above means ‘to eat’ written in which language?**
   1. **Finnish**
   2. **Swedish**

**
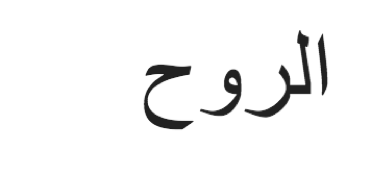
**

1. **The above means ‘soul’ written in which language?**
   1. **Arabic**
   2. **Armenian**

**
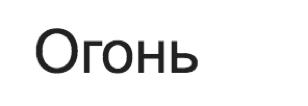
**

1. **The above means ‘fire’ written in which language?**
   1. **Russian**
   2. **Bulgarian**

**WEIGHT ESTIMATION**

1. **A tennis ball weighs:**
   1. **58.5g**
   2. **5.85g**
2. **What does a typical (class 1A) fire extinguisher weigh?**
   1. **115kg**
   2. **1.15kg**
3. **Which weighs more, on average?**
   1. **Apricot**
   2. **Avocado**
4. **Which weighs more, on average?**
   1. **An ostrich egg**
   2. **A hen’s egg**
5. **What does an average baking potato weigh?**
   1. **18 g**
   2. **180g**

**
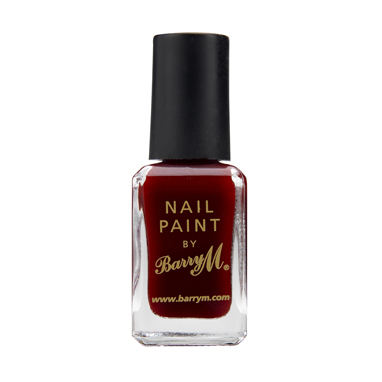
**

1. **An average pot of nail varnish weighs:**
   1. **62g b. 162 g**

**
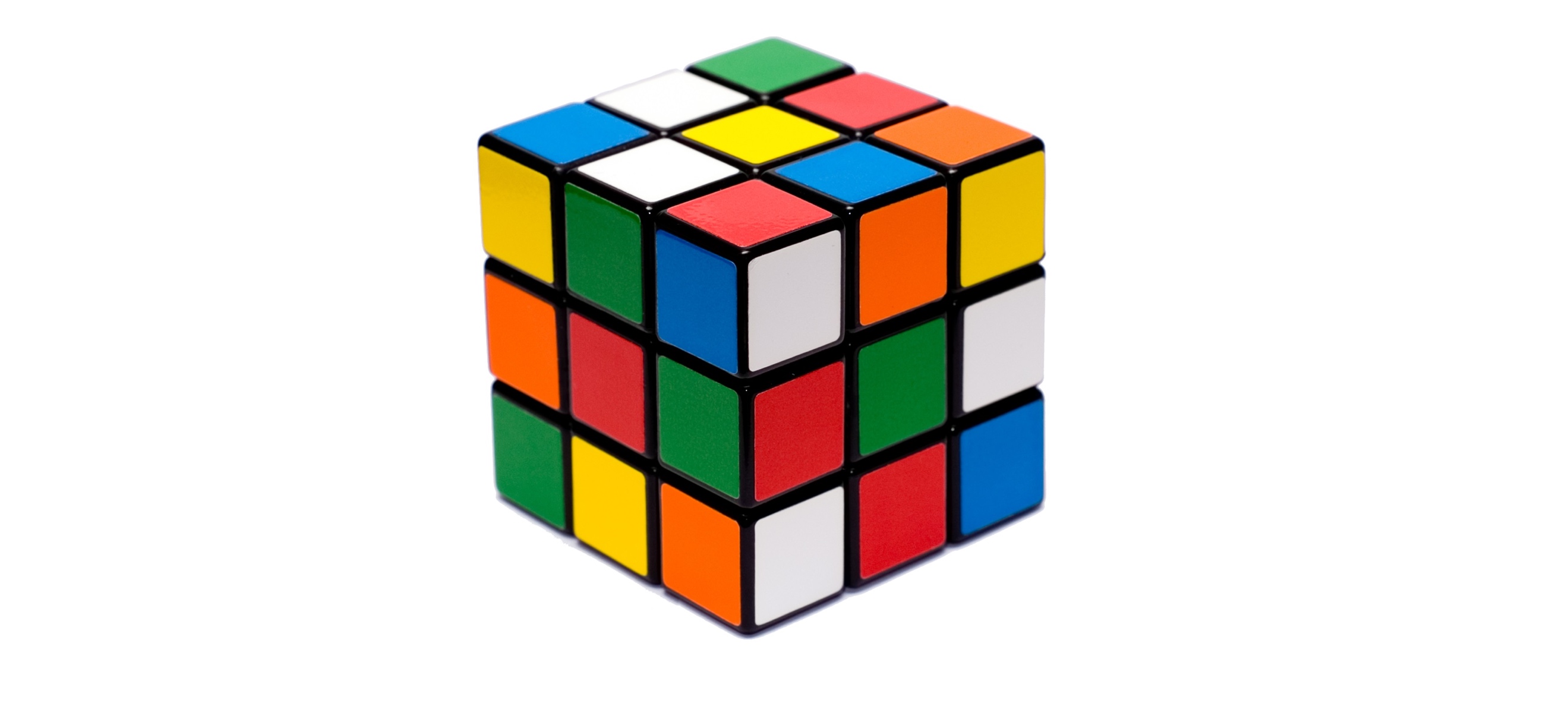
**

1. **A Rubik’s cube weighs:**
   1. **0.14kg b. 1.14kg**

**
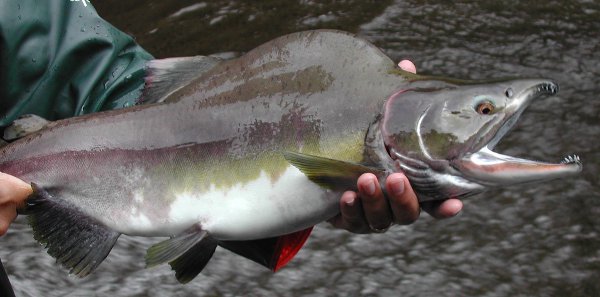
**

1. **An average Pink Salmon weighs:**
   1. **1.7kg b. 17kg**

**
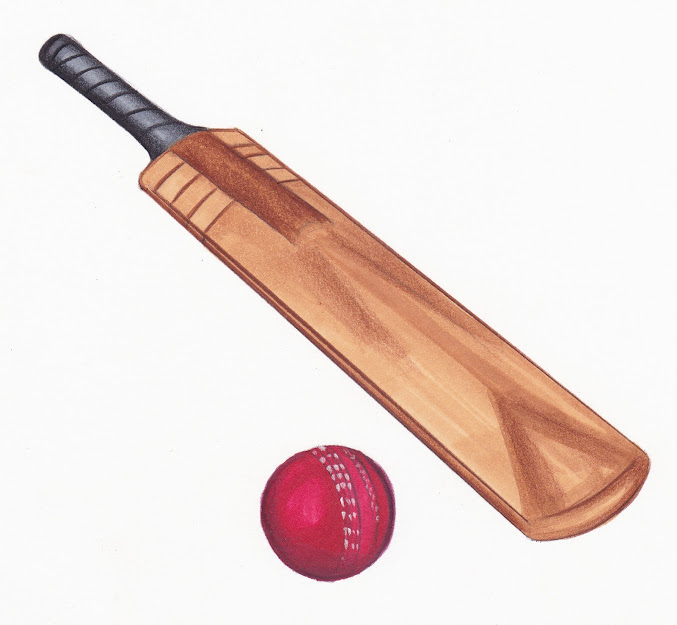
**

1. **A cricket bat weighs:**
   1. **1.4 kg**
   2. **14 kg**

**
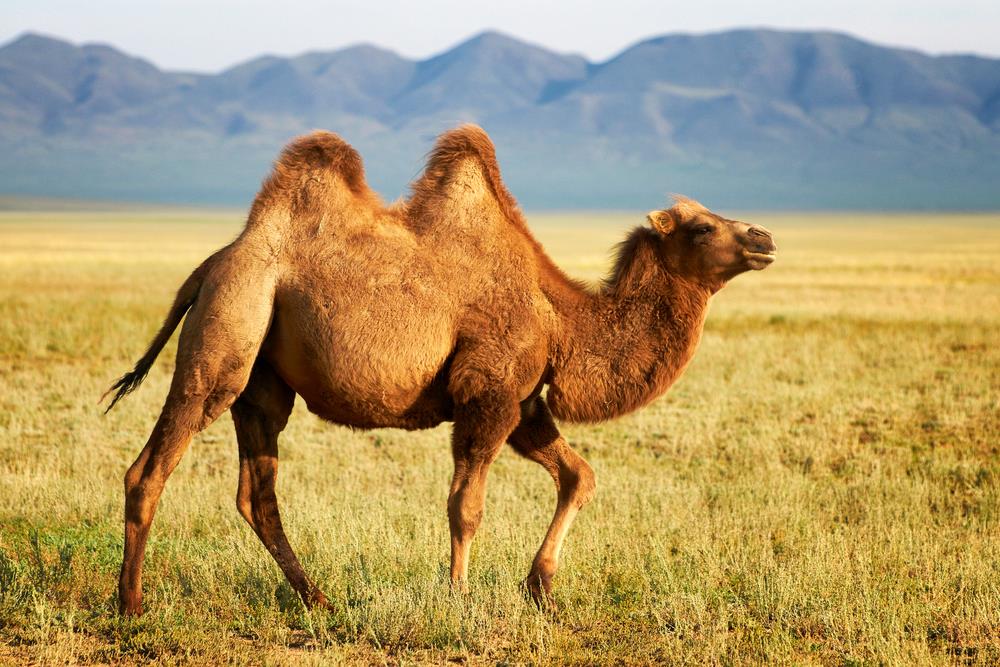
**

1. **The average weight of a camel is:**
   1. **480kg**
   2. **48kg**

**Estimated score (out of 40): __________**

**YOUR ID NUMBER: __________ THEIR ID NUMBER: ___________**

Please rate this person according to the following scale:

**1------------2------------3------------4------------5------------6------------7**

**Not At All/ NOT TRUE**

**Somewhat**

**Very Much/ TRUE**

1. Members of your group respect and admire them _____
2. Members of your group do NOT want to be like them_____
3. They enjoy having control over other members of the group_____
4. Members of your group always expect them to be successful_____
5. They often try to get their own way regardless of what others in the group may want_____
6. Members of your group do NOT value their opinion_____
7. They are willing to use aggressive tactics to get their way_____
8. They are held in high esteem by members of the group_____
9. They try to control others rather than permit others to control them_____
10. They do NOT have a forceful or dominant personality_____
11. Members of the group know it is better to let them have their way_____
12. They do NOT enjoy having authority over other members of the group_____
13. Their unique talents and abilities are recognized by others in the group_____
14. They are considered an expert on some matters by members of the group_____
15. Members of your group seek their advice on a variety of matters_____
16. Members of your group are afraid of them_____
17. Others do NOT want to spend time with them_____

**During the group quiz discussion, this person:**

1. Had high status_____
2. Led the task_____
3. Was paid attention_____

**Overall:**

1. I like this person _____ 22. I like working with this person________

| \| **Group no.** \| Group_Affiliation \| **Date of participation** \| \| --- \| --- \| --- \| \| *1* \| PILOT TEST (Office) --**DELETED** \| 22/05/2017 \| \| 2 \| Newquay Zoo Staff \| 20/07/2017 \| \| 3 \| FXPlus Library Staff \| 15/08/2017 \| \| 4 \| Falmouth Cruiseship Ambassadors_4 \| 15/08/2017 \| \| 5 \| Falmouth Cruiseship Ambassadors_5 \| 15/08/2017 \| \| 6 \| REDWING Community Centre \| 11/10/2017 \| \| 7 \| REDWING_2 \| 11/10/2017 \| \| 8 \| SourceFM Presenters \| 22/10/2017 \| \| 9 \| Nankersey Male Choir \| 07/11/2017 \| \| 10 \| Active Plus_Hayle \| 04/12/2017 \| \| 11 \| Active Plus_2 -**DELETED FOR WITHIN-GROUP** \| 04/12/2017 \| \| 12 \| Flushing & Mylor Gig Rowing \| 06/12/2017 \| \| 13 \| SSE_Truro Health&Wellbeing Innovation Centre \| 15/01/2018 \| \| 14 \| ActivePlus_St Ives \| 16/01/2018 \| \| 15 \| Active Plus_Clies \| 17/01/2018 \| \| 16 \| Psych-Soc_Streatham \| 30/01/2018 \| \| 17 \| Psych-Soc_Streatham2 \| 30/01/2018 \| \| 18 \| Dyslexia Cornwall_Truro Innovation Centre \| 31/01/2018 \| \| 19 \| Bio-Soc Streatham_3 \| 08/02/2018 \| \| 20 \| Streatham Students_4 \| 08/02/2018 \| \| 21 \| Clarinet Choir \| 08/02/2018 \| \| 22 \| Hotel Perfect_Tremough Innovation Centre \| 09/02/2018 \| \| 23 \| Falmouth Kayak Club_students \| 13/02/2018 \| \| 24 \| Roaming CIC _Penzance \| 15/02/2018 \| \| 25 \| Penryn MSc students \| 26/02/2018 \| \| 26 \| Mylor Mums \| 04/03/2018 \| \| 27 \| Philosopher's Hat \| 05/03/2018 \| \| 28 \| Calstock Chess Club \| 06/03/2018 \| \| 29 \| Falmouth Fish Sea Shanty Collective \| 07/03/2018 \| \| 30 \| Pirates of Penryn boardgame creators \| 07/03/2018 \| \| 31 \| Cambourne Chess Club \| 12/03/2018 \| \| 32 \| Penryn MSc students_2 \| 15/03/2018 \| |
| --- | --- | --- | --- | --- | --- | --- | --- | --- | --- | --- | --- | --- | --- | --- | --- | --- | --- | --- | --- | --- | --- | --- | --- | --- | --- | --- | --- | --- | --- | --- | --- | --- | --- | --- | --- | --- | --- | --- | --- | --- | --- | --- | --- | --- | --- | --- | --- | --- | --- | --- | --- | --- | --- | --- | --- | --- | --- | --- | --- | --- | --- | --- | --- | --- | --- | --- | --- | --- | --- | --- | --- | --- | --- | --- | --- | --- | --- | --- | --- | --- | --- | --- | --- | --- | --- | --- | --- | --- | --- | --- | --- | --- | --- | --- | --- | --- | --- | --- | --- |
|  |

| \| name \| position \| \| --- \| --- \| \| A**** ***** \| Mother \| \| Andrew \| Councillor \| \| Andrew **** \| Councillor \| \| Andrew ***** \| Broadcaster \| \| ***** ***** \| VC of Falmouth Uni \| \| Arthur **** \| Union leader \| \| B. Portman \| X Mayor \| \| Ben Bradshaw \| MP \| \| B** ***** \| Head Teacher \| \| Beppe Grillo \| Politician \| \| Beyonce \| Singer \| \| Bob \|  \| \| Bob Devereux \| Poet, Playwrite \| \| Boris Johnson \| Foreign Secretary \| \| Boris Johnson \| Foreign Secretary \| \| Boris Johnson \| Foreign Secretary \| \| Boris Johnson \| Foreign Sec \| \| Boris Johnson \| F. minister \| \| Boris Johnson \| Foreign Minister \| \| Brad Brown \| Creator of "more to life" programme \| \| Brother Roger \| Late Friar of Taize \| \| Captain Holt \| Captain of Police (Brooklyn 99) \| \| Carl Sagan \| SETI, attack on superstition, novel Contact \| \| Caroline Lucas \| Leader Green Party \| \| Christopher ***** \| Director of******, Cathedral \| \| Christopher ***** \| Lead story developer \| \| Colin ****** \| Neighbour \| \| Cornelius Oliver \| Councillor \| \| Cyndi Lauper \| Singer/ human rights activist \| \| David Attenborough \| Broadcaster \| \| David Attenborough \| Broadcaster \| \| David Attenborough \| Tv presenter \| \| David Attenborough \| Conservationist/presenter \| \| David Attenborough \| BBC Presenter \| \| David Attenborough \| tv presenter/environmentalist/wildlife activist \| \| David Attenborough \| Presenter \| \| David Attenborough \| Presenter/ecologist \| \| David Attenborough \| Naturalist \| \| David Attenborough \| Tv presenter \| \| Deborah Francis-White \| Leads the Guilty Feminist Podcast \| \| Dennis Skinner \| MP For Bolsever \| \| Donald Trump \| Prime Minister \| \| Dr Nigel Hewitt \| Director of a Charity \| \| Dr Peter Rodgers \| GP/chairman of tennis club \| \| Eddie Calvo \| Govenor of Guam \| \| Eddie Home \| Football Club Manager \| \| Edward Davey \| MP \| \| Enoch Powell \| Politician \| \| Frank Whittal \| Inventor \| \| Friedensreich Hundertwasser \| Artist \| \| George Eustace \| MP \| \| George Orwell \| Author \| \| Ghandi \| leader \| \| Grayson Perry \| Artist/potter \| \| Grenville Chapple \| Mayor \| \| Henry Bew \| Head of English Department \| \| Jamie Shiner \| Business Leader \| \| Jane Goodall \| Primatologist \| \| Jenny \| Artist/musician \| \| Jeremy Corbyn \| Leader of opposition \| \| Jeremy Corbyn \| Politician \| \| Jeremy Corbyn \| Labour Party Leader \| \| Jeremy Corbyn \| Leader Labour Party \| \| Jeremy Corbyn \| Labour Party Leader \| \| Jeremy Corbyn \| Leader of the labour party \| \| Jeremy Corbyn \| Labour Leader \| \| Jeremy Corbyn \| labour Party Leader \| \| Jeremy Corbyn \| Labour Party Leader \| \| Jeremy Corbyn \| Leader of the opposition \| \| Jeremy Corbyn \| Leader of the labour party \| \| Jeremy Corbyn \| Leader of opposition \| \| Jeremy Hunt \| Health Cabinet \| \| JK Rowling \| Author \| \| Jo Feather \| Taught sailing after the war, got MBE \| \| John Goodman \| Newquay Facebook Community Admin \| \| John Pollard \| Councillor \| \| Johnny Depp \| Celebrity \| \| Lesley \| manageress of Hayle community centre \| \| L***** ****** \| Day care centre manager \| \| Linda \| Mayor \| \| Mancelo Rebelo Sousa \| Republic President \| \| Marconi For Radio \| Early Communications \| \| Margaret Thatcher \| Prime Minister \| \| Mark Ashton \| Activist \| \| Mary Berry \| Cook \| \| Mary Holborough \| High Sherrif \| \| Michael Gove \| M.P. \| \| Michael Tyade \| Musician \| \| M***** ******* \| Step Father \| \| Mrs May \| Prime Minister \| \| Mrs Sanderson \| Headmistress of local school \| \| Mrs x \| Headteacher of primary school \| \| na \| na \| \| Nicholas ******* \| Leader of local group \| \| Nicolae Ceausescu \| President (Former) \| \| Oscar Wilde \| author/activist/dandy \| \| Ozzy Osbourne \| Rock Star Supreme \| \| Patrick Gale \| Author \| \| Paul \| International project director \| \| Penny HC Dinh \| Youtuber \| \| Peter L Mere \| Peace Campaigner CND \| \| Phil ***** \| Traveller&Gypsy Liason Officer \| \| Prince Charles \| Duke of Cornwall \| \| Prince William \| Heir to the Throne \| \| The Queen \| Head of State \| \| The Queen \| Queen \| \| Richard Charter \| ex Bishop of London \| \| ****** ****** \| Chess club secretary + local accountant \| \| Richard ****** \| free software advocate & software developer \| \| Rick Stein \| Celeb Chef \| \| R********** \| President of ****** Association \| \| Rodrigo Duterte \| President of Phillipines \| \| Roselyn \| Director \| \| Rupaul Charles \| Drag Queen \| \| Sam Mezec \| States of Jersey Reform Party Member \| \| Sarah Newton \| MP Falmouth \| \| Sarah Newton \| M.P. \| \| Sarah Newton \| Tory MP Truro & Falmouth \| \| Sarah Porter \| Councilor \| \| Shanne Sands \| Poet \| \| ******** \| Head drama teacher sixth form \| \| Sheryll Murray \| Local MP \| \| Shinzo Abe \| Prime minister of Japan \| \| Sir Humphry Davy \| Statue \| \| Steve ***** \| Advocacy Support Worker \| \| Tarja Halona \| former president of Finland \| \| Theresa May \| The Prime Minister \| \| The Queen \| Queen of England \| \| The Queen \| Monarch \| \| Theresa May \| Prime Minister \| \| Theresa May \| Prime Minister \| \| Theresa May \| PM \| \| Theresa May \| Prime Minister \| \| Theresa May \| Prime minister \| \| Theresa May \| Prime Minister \| \| Theresa May \| Prime Minister \| \| Theresa May \| Prime minister \| \| Theresa May \| Prime Minister \| \| Theresa May \| Prime Minister \| \| Theresa May \| Prime Minister \| \| Theresa May \| Prime Minister \| \| Theresa May \| Prime Minister \| \| Theresa May \| Prime Minister \| \| Theresa May \| Prime Minister \| \| theresa May \| Prime minister \| \| Theresa May \| Prime Minister \| \| Theresa May \| Prime Minister \| \| Theresa May \| Prime Minister \| \| Theresa May \| Prime Minister \| \| Tony Blair \| past PM \| \| Traian Bruma \| Head of alternative university in Romania \| \| Vicky M \| Green politician \| \| Winston Churchill \| Wartime PM \| \| Winston Churchill \| Prime Minister \| \| Winston Churchill \| ex Prime minister \| |
| --- | --- | --- | --- | --- | --- | --- | --- | --- | --- | --- | --- | --- | --- | --- | --- | --- | --- | --- | --- | --- | --- | --- | --- | --- | --- | --- | --- | --- | --- | --- | --- | --- | --- | --- | --- | --- | --- | --- | --- | --- | --- | --- | --- | --- | --- | --- | --- | --- | --- | --- | --- | --- | --- | --- | --- | --- | --- | --- | --- | --- | --- | --- | --- | --- | --- | --- | --- | --- | --- | --- | --- | --- | --- | --- | --- | --- | --- | --- | --- | --- | --- | --- | --- | --- | --- | --- | --- | --- | --- | --- | --- | --- | --- | --- | --- | --- | --- | --- | --- | --- | --- | --- | --- | --- | --- | --- | --- | --- | --- | --- | --- | --- | --- | --- | --- | --- | --- | --- | --- | --- | --- | --- | --- | --- | --- | --- | --- | --- | --- | --- | --- | --- | --- | --- | --- | --- | --- | --- | --- | --- | --- | --- | --- | --- | --- | --- | --- | --- | --- | --- | --- | --- | --- | --- | --- | --- | --- | --- | --- | --- | --- | --- | --- | --- | --- | --- | --- | --- | --- | --- | --- | --- | --- | --- | --- | --- | --- | --- | --- | --- | --- | --- | --- | --- | --- | --- | --- | --- | --- | --- | --- | --- | --- | --- | --- | --- | --- | --- | --- | --- | --- | --- | --- | --- | --- | --- | --- | --- | --- | --- | --- | --- | --- | --- | --- | --- | --- | --- | --- | --- | --- | --- | --- | --- | --- | --- | --- | --- | --- | --- | --- | --- | --- | --- | --- | --- | --- | --- | --- | --- | --- | --- | --- | --- | --- | --- | --- | --- | --- | --- | --- | --- | --- | --- | --- | --- | --- | --- | --- | --- | --- | --- | --- | --- | --- | --- | --- | --- | --- | --- | --- | --- | --- | --- | --- | --- | --- | --- | --- | --- | --- | --- | --- | --- | --- | --- | --- | --- | --- | --- | --- | --- | --- | --- | --- | --- | --- | --- | --- | --- | --- | --- | --- | --- | --- | --- | --- | --- | --- | --- | --- | --- |
|  |

| \| **name** \| **want to learn:** \| \| --- \| --- \| \| Alex Pritchard \| Politics \| \| Alf Coles \| Devloping conviction in others \| \| Alice \| social skills \| \| Alistair \| Maths education \| \| Amanda Brown \| Yoga Skills \| \| Andrew George \| Clear public speaking and articulation \| \| Andrew Mitchell \| Council affairs \| \| Andrew Mitchell \| Council affairs \| \| Andrew Nancharrow \| Wooden Boat Building \| \| Andrew Tozer \| Artist \| \| Annabelle Waite \| Singing/performance \| \| Augustine \| Rhetoric \| \| B. Portman \| ability to sort thngs out \| \| Ben Mayer \| na \| \| Bethany Saunders \| See life like she does \| \| Bobby Moore \| Football Skills \| \| Boris Johnson \| Expand on knowledge of German \| \| Boris Johnson \| Foreign Sec. \| \| Brian Cox \| Scientific Knowledge \| \| Brian Goldthorp \| Knowledge \| \| Bryony Oncuul \| Their knowledge of colonisation and heritage \| \| Carl Hester \| Dressage coaching \| \| Caroline Harris/boss \| eventing/riding \| \| Caroline Lucas \| Eloquence \| \| Che Willbraham \| Digital Story making \| \| Chris D \| Patience/teaching skills \| \| Chris Rubbery \| Advance Sailing Safety \| \| Churchill \| Public Speaking \| \| Claire Hall \| sewing skills \| \| Claire Ingleheart \| Personality&Music \| \| Cliff Thomas \| His philosophy on life \| \| College professor \| communication skills \| \| Dalai Lama \| Wisdom \| \| Dali Lama \| Tolerance \| \| Dan \| To play music/sing well \| \| Darryl Thorpe \| Seal Rescue \| \| David Attenborough \| Animal knowledge \| \| David Attenborough \| to inspire and motivate \| \| David Bowie \| Knowledge \| \| DAVID COLEMAN \| DEMOGRAPHY \| \| Dawn French \| Comedy \| \| Delia Smith \| Cooking \| \| Derek Guthrie \| Knowledge of art \| \| Doctor \| Patience \| \| Dr Drummond \| be like him \| \| Dr. Phil Hammond \| Presentaiton & speaking skills \| \| Eddy Jones \| na \| \| Elaine Taneye \| Musical understanding & technique \| \| Emilia Clarke \| Confidence \| \| Emma Watson \| Influential Speaking on feminist issues \| \| Eniola Aluko \| Football \| \| Eric Meijaard \| Productivity, influence \| \| Fiona Richardson \| Positivity & Passion \| \| Frank Auerbach \| Painting \| \| Fuzz Townsend \| Mechanical Knowledge/car restoration \| \| Gary \| Be awesome \| \| Gary Ward (senior bird keeper) \| knowledge of bird husbandry \| \| Giorgio Fraticelli \| outgoing/ networking skills \| \| Grayson Perry \| artistic confidence \| \| Hannah \| choreography \| \| Head horse worker, Bosence Farm \| Equine Therapy \| \| Helen Mirren \| to be confident & forthright \| \| Helen Porter \| Musical performance \| \| J. K. Rowling \| Success \| \| J.K. Rowling \| writing skills \| \| James Scourse \| ability to understand and use knowledge \| \| Jamie Oliver \| How to cook \| \| Jane Sand \| How to paint \| \| Jard Acton \| Pleasant Nature Outdoor Sessional Skills \| \| Jennifer Morrison \| Acting Skills \| \| Jeremy Corbyn \| Conviction \| \| Jeremy Paxman \| General Knowledge \| \| JK Rowling \| writing \| \| JK Rowling \| Writing \| \| John \| Zen Buddhism \| \| John Gordon \| Poet \| \| John Gordons \| Guitar Teacher \| \| John Williams \| Guitar Skills \| \| Johnny Cowling \| Ability to make people laugh \| \| Julia Nott \| Play piano better \| \| Kerstin Skining \| organisation \| \| Kim Conchie(Cornwall Chamber of Commerce) \| Management & Presentation Skills \| \| Lawrence John \| Tailoring \| \| Lecturer X \| Enthusiasm & public speaking \| \| Les \|  \| \| Lesley Chenels \| Fairness \| \| Leslie Bradley Peers \| Standing my ground, networking, communicating \| \| Lisa Wisdom \| Artistic forwardness/success \| \| M Bryant \| Outlook on life \| \| Madhar Jaffrey \| Recipes, Cooking, techniques and skills \| \| Margaret Attwood \| Writing \| \| Marianne De Trey \| Living a potter's life \| \| Mary \| Only see the best in people \| \| Mary Chan \| their profession \| \| Matthew Mercer \| Creativity \| \| Matthew Rogers \| To relax \| \| Matthew Rogers \| Management skills \| \| Max Shepherd \| most things \| \| Melanie Wheeler \| be more like them \| \| Mestre zo Antonio \| Caspiera \| \| Michael Smith \| Diplomacy \| \| Mike Parknoy \| Drumming & Music \| \| Mom \| Perseverance, energetic personality, wanting to step out of comfort zone \| \| Mother \| patience/compassion \| \| Mr Harvey \| Biology \| \| Mum \| kindness, patience, understanding \| \| Music band \| strength of will \| \| My Mother \| loyalty, resiliance \| \| na \| na \| \| na \| na \| \| Nicholas Smith \| How to lead others \| \| Nicole Crane \| Quilting \| \| Nik Elvy \| How to lead/teach/manage youth group \| \| Niki Pearce \| Socialising Skills \| \| no.127 \| excellent parenting skills \| \| Olivia A \| Dance \| \| Oscar Wilde \| confidence/writing skills \| \| Pat Parelli \| Horsemanship \| \| Paul Smith \| Motivation \| \| Peter Jones \| Business acumen \| \| Phil \| Carpentry/ ship building \| \| Prince William \| Modern way of thinking \| \| Puzzle \| Painting Landscapes \| \| Queen Elizabeth \| na \| \| Queen Elizabeth II \| her patience \| \| Richard Baker \| Book keeping skills \| \| Richard Branson \| Business acumen \| \| Richard Osmond \| General Knowledge \| \| Robert Fischer \| Chess Genius \| \| Roisin Perry \| Dedication \| \| Ros Williams \| Printmaking \| \| Roselyne \| Management \| \| Roxana Marin \| enthusiasm & energy \| \| S. Thompson \| Patience \| \| Sean \| Paddleboarding weSUP Gylly Beach \| \| Shakleton \| Adventurous spirit/exploration \| \| Shane Glachin \| Academic \| \| Shannon McManus \| music \| \| Shirley Williams \| Leadership/negotiation \| \| Simon Clark \| Video editing skills \| \| Simon Parry \| Knowledge \| \| Stan Lee \| Drawing Style \| \| Stephen Hawking \| Cosmology \| \| Suvi Pattibagar \| Discipline & organisational skills \| \| Suzanne Cotter \| Knowledge \| \| Terry McClintock \| Indian Cooking \| \| The PM \| general skills \| \| Tim Brabants \| commitment to training \| \| Tim Shaw \| Sculptor \| \| Tom Rice \| His knowledge of anthropology \| \| Tony Blair \| Self-confidence \| \| Wilfred Owen \| Skills with words \| \| Will Smith \| Ambition \| \| William F Sanday \| Music, oragn & piano \| \| Yo yo ma \| technique \| |  |
| --- | --- | --- | --- | --- | --- | --- | --- | --- | --- | --- | --- | --- | --- | --- | --- | --- | --- | --- | --- | --- | --- | --- | --- | --- | --- | --- | --- | --- | --- | --- | --- | --- | --- | --- | --- | --- | --- | --- | --- | --- | --- | --- | --- | --- | --- | --- | --- | --- | --- | --- | --- | --- | --- | --- | --- | --- | --- | --- | --- | --- | --- | --- | --- | --- | --- | --- | --- | --- | --- | --- | --- | --- | --- | --- | --- | --- | --- | --- | --- | --- | --- | --- | --- | --- | --- | --- | --- | --- | --- | --- | --- | --- | --- | --- | --- | --- | --- | --- | --- | --- | --- | --- | --- | --- | --- | --- | --- | --- | --- | --- | --- | --- | --- | --- | --- | --- | --- | --- | --- | --- | --- | --- | --- | --- | --- | --- | --- | --- | --- | --- | --- | --- | --- | --- | --- | --- | --- | --- | --- | --- | --- | --- | --- | --- | --- | --- | --- | --- | --- | --- | --- | --- | --- | --- | --- | --- | --- | --- | --- | --- | --- | --- | --- | --- | --- | --- | --- | --- | --- | --- | --- | --- | --- | --- | --- | --- | --- | --- | --- | --- | --- | --- | --- | --- | --- | --- | --- | --- | --- | --- | --- | --- | --- | --- | --- | --- | --- | --- | --- | --- | --- | --- | --- | --- | --- | --- | --- | --- | --- | --- | --- | --- | --- | --- | --- | --- | --- | --- | --- | --- | --- | --- | --- | --- | --- | --- | --- | --- | --- | --- | --- | --- | --- | --- | --- | --- | --- | --- | --- | --- | --- | --- | --- | --- | --- | --- | --- | --- | --- | --- | --- | --- | --- | --- | --- | --- | --- | --- | --- | --- | --- | --- | --- | --- | --- | --- | --- | --- | --- | --- | --- | --- | --- | --- | --- | --- | --- | --- | --- | --- | --- | --- | --- | --- | --- | --- | --- | --- | --- | --- | --- | --- | --- | --- | --- | --- | --- | --- | --- | --- | --- | --- | --- | --- | --- | --- | --- | --- | --- | --- | --- | --- | --- |
|  |  |
|  |  |
